# Significantly Enhanced Energy Storage Density by Modulating the Aspect Ratio of BaTiO_3_ Nanofibers

**DOI:** 10.1038/srep45179

**Published:** 2017-03-23

**Authors:** Dou Zhang, Xuefan Zhou, James Roscow, Kechao Zhou, Lu Wang, Hang Luo, Chris R. Bowen

**Affiliations:** 1 State key Laboratory of Powder Metallurgy, Central South University, Changsha, Hunan 410083, China; 2 Department of Mechanical Engineering, University of Bath, Bath, BA2 7AY, UK; 3 College of Chemistry and Chemical Engineering, Central South University, Changsha 410083, China

## Abstract

There is a growing need for high energy density capacitors in modern electric power supplies. The creation of nanocomposite systems based on one-dimensional nanofibers has shown great potential in achieving a high energy density since they can optimize the energy density by exploiting both the high permittivity of ceramic fillers and the high breakdown strength of the polymer matrix. In this paper, BaTiO_3_ nanofibers (NFs) with different aspect ratio were synthesized by a two-step hydrothermal method and the permittivity and energy storage of the P(VDF-HFP) nanocomposites were investigated. It is found that as the BaTiO_3_ NF aspect ratio and volume fraction increased the permittivity and maximum electric displacement of the nanocomposites increased, while the breakdown strength decreased. The nanocomposites with the highest aspect ratio BaTiO_3_ NFs exhibited the highest energy storage density at the same electric field. However, the nanocomposites with the lowest aspect ratio BaTiO_3_ NFs achieved the maximal energy storage density of 15.48 J/cm^3^ due to its higher breakdown strength. This contribution provides a potential route to prepare and tailor the properties of high energy density capacitor nanocomposites.

High permittivity materials have received significant attention in recent years due to their potential for application in high energy density capacitors; these are a key technology for the development of portable electronic devices, stationary power systems and high power microwave systems^1^,^2^,^3^,^4^,^5^. Ferroelectric ceramics such as lead zirconate titanate (PZT) and barium titanate (BaTiO_3_) are the preferred choice for application in capacitors because of their high permittivity^6^,^7^,^8^. In general, the energy density (*U*_e_) of dielectric materials is defined as the integral *U*_e_ = ∫ *E*d*D*, where *E* is the electric field and *D* is the electric displacement^9^,^10^. Therefore, a high breakdown strength is a key factor to obtain a high energy density, and high permittivity ferroelectric ceramics often exhibit a relatively low dielectric strength and high dielectric loss which limits the energy density^11^,^12^.

In recent decades, an number of efforts have been made to fabricate ceramics/polymer nanocomposites to overcome this limitation^13^,^14^,^15^. The nanocomposite systems combine the high permittivity of ceramics with the high breakdown strength, low dielectric loss, and mechanical flexibility of polymers. To achieve a high energy density, researchers have developed a variety of approaches to improve the dielectric properties of nanocomposites. For example, (i) the use of high permittivity polymer matrixes such as polyvinylidene fluoride (PVDF) and its copolymers to reduce the contrast of dielectric properties between ceramic nanoparticles and polymer matrixes^16^,^17^,^18^ and (ii) chemical modification on the surface of ceramic nanoparticles to enhance nanoparticle dispersion in the polymer matrix and improve interfacial adhesion between matrix and particle^19^,^20^,^21^.

In addition, recent studies have demonstrated that the morphology and geometry of ceramic nanoparticles influence the dielectric properties of nanocomposites^22^,^23^,^24^. For 0–3 type ceramic-polymer nanocomposites, where spherical zero-dimensional ceramic nanoparticles are embedded in a three-dimensionally connected polymer matrix, a high volume fraction (>50 vol%) of nanoparticles is necessary to achieve a high permittivity nanocomposite. However, this can lead to a reduced breakdown strength and low mechanical flexibility^25^. For 1–3 type ceramic-polymer nanocomposites, where one-dimensional ceramic nanoparticles are dispersed in a three-dimensionally connected polymer matrix, a number of studies have indicated that high aspect ratio ceramic fillers can improve the dielectric properties and energy density of nanocomposites more efficiently compared to spherical ceramic fillers^26^,^27^,^28^. Andrews *et al*. developed a micromechanics approach and finite element models to study the effect of ceramic filler aspect ratio on the electro-elastic properties of nanocomposites^29^. The results showed the electromechanical coupling can increase up to 60 times compared to its initial values when the aspect ratio was increased from 1 to 10 at 30 vol % of ceramic filler. Tang *et al*. demonstrated that nanocomposites based on high aspect ratio PZT nanowires exhibited an increased energy density, which was 77.8% higher than lower aspect ratio PZT nanowires^30^.

In this study, we have prepared P(VDF-HFP) nanocomposites with a range of aspect ratio BaTiO_3_ nanofibers (NFs) synthesized by a two-step hydrothermal method. There have been a variety of reported methods for synthesizing BaTiO_3_ NFs, such as the two-step hydrothermal method, one-step hydrothermal method, topochemical solid-state reaction and anodic aluminum oxide template method^25^,^31^,^32^,^33^. The two-step hydrothermal method has attracted attention due to its superiority in terms of the synthesis of single crystalline nanofibers, morphology control, homogeneity at the molecular level, low temperature processing and simple experimental approach. The effects of aspect ratio and volume fraction of the BaTiO_3_ NFs on the dielectric constant (relative permittivity), breakdown strength and energy storage density of the nanocomposites were investigated systematically. On increasing the BaTiO_3_ NF volume fraction or aspect ratio, the dielectric constant and maximum electric displacement of the nanocomposites increased monotonically while the breakdown strength decreased monotonically. Under the same electric field, the nanocomposites with higher volume fraction or higher aspect ratio BaTiO_3_ NFs possessed higher discharged energy density. The maximal energy storage density reached 15.48 J/cm^3^ in nanocomposites containing 7.5 vol% BaTiO_3_ NFs synthesized at 210 °C for 2 h under the electric field of 300 kV/mm.

## Results and Discussion

**Figure 1:**
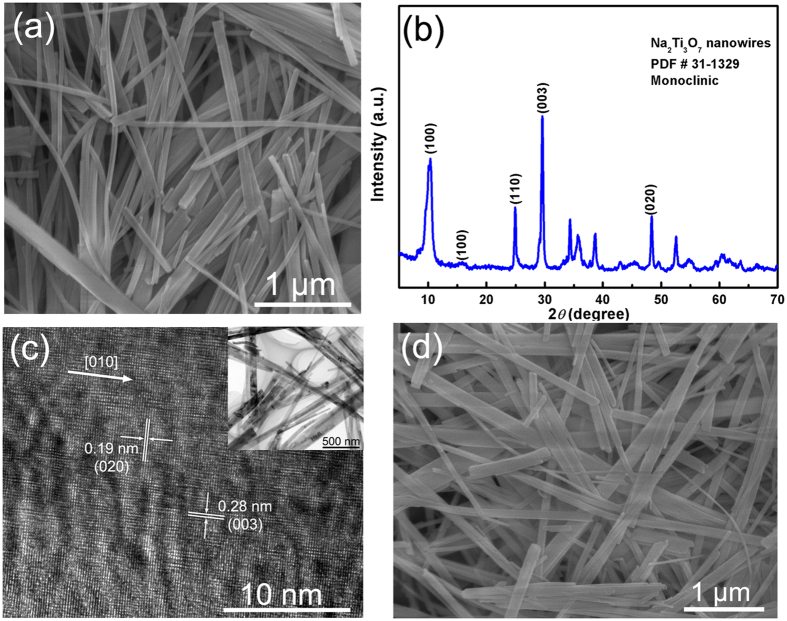
(**a**) SEM image, (**b**) XRD pattern and (**c**) TEM images of hydrothermally synthesized Na_2_Ti_3_O_7_ nanofibers (NT NFs). (**d**) SEM image of H_2_Ti_3_O_7_ NFs.

In order to obtain BaTiO_3_ nanofibers (BT NFs), an initial hydrothermal reaction was used to synthesize Na_2_Ti_3_O_7_ nanofibers (NT NFs) due to its extensive research history and easily controlled nanofiber morphology^25^. Figure 1a shows the morphology of hydrothermally synthesized Na_2_Ti_3_O_7_ nanofibers (NT NFs) which exhibited a high aspect ratio and favorable dispersibility. Figure 1b shows the XRD pattern of NT NFs indexed to monoclinic Na_2_Ti_3_O_7_ (PDF card NO. 31–1329). The corresponding TEM images were shown in Fig. 1c. The NT NFs possessed a smooth surface with a diameter of approximately 100 nm. The clear lattice fringes shown in Fig. 1c demonstrate that the NT NFs were single-crystalline. The parallel lattice spacings were approximately 0.19 nm and 0.28 nm, which correspond to the (020) and (003) planes, respectively, and reveal that the NT NFs grew in the [010] direction. Figure 1d shows the SEM image of H_2_Ti_3_O_7_ nanofibers (HT NFs), which retained the morphology of the Na_2_Ti_3_O_7_ nanofibers.

**Figure 2:**
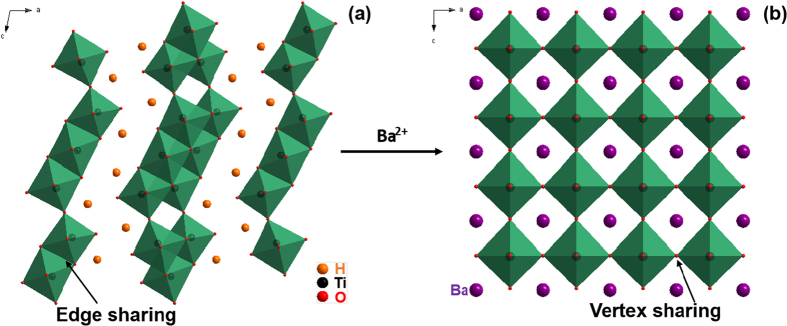
Schematic diagrams of the crystalline structures of (**a**) H_2_Ti_3_O_7_ and (**b**) BaTiO_3_.

The HT NFs were transformed to BaTiO_3_ NFs by a second hydrothermal reaction. The H_2_Ti_3_O_7_ phase is a layered titanate, which is a good precursor for soft chemical synthesis because of its open structure that enables ion exchange and topochemical transformation. Figure 2a,b shows schematic diagrams of the crystalline structures of H_2_Ti_3_O_7_ and BaTiO_3_, respectively. During the second hydrothermal reaction, the Ba^2+^ ions diffuse into the host lattice of H_2_Ti_3_O_7_ by ion exchange with H^+^, which leads to a rearrangement of the octahedra sharing and structural transformation to perovskite BaTiO_3_. The Ba^2+^ ions possess a higher positive charge compared with H^+^, thereby promoting the structural transformation. In addition, it is known that the edge sharing octahedra were driven into vertex sharing octahedra during the topochemical transformation^34^. Therefore, the HT NFs were successfully transformed into BaTiO_3_ NFs.

**Figure 3:**
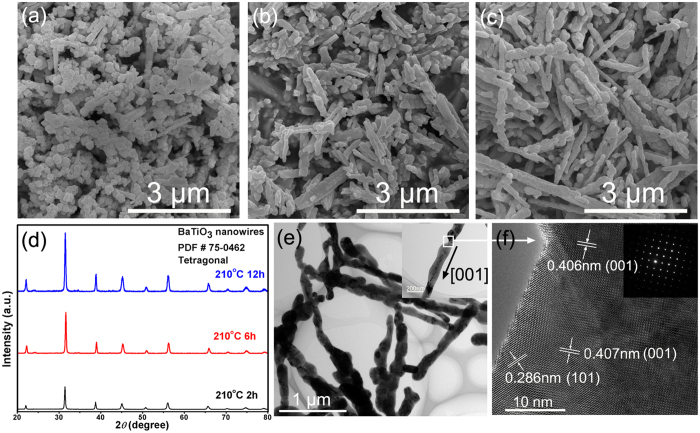
SEM images of BaTiO_3_ NFs hydrothermally synthesized at 210 °C for (**a**) 2 h, (**b**) 6 h, (**c**) 12 h. (**d**) XRD patterns of BaTiO_3_ NFs. (**e**) TEM images of BaTiO_3_ NFs synthesized at 210 °C for 12 h. (**f**) HRTEM image of a typical BaTiO_3_ NF and corresponding SADP.

Figure 3a–c show the sizes and morphologies of BaTiO_3_ NFs hydrothermally synthesized at 210 °C for 2–12 h, respectively. The corresponding XRD patterns are shown in Fig. 3d. The diffraction patterns indicate that tetragonal BaTiO_3_ (PDF card NO. 75–0462) without any impurity phase can be obtained at 210 °C with a reaction time ranging from 2 h to 12 h and the crystallization of BaTiO_3_ was improved at longer reaction times. When the reaction time was 2 h, the products were a mass of nanoparticles and few nanofibers, as shown in Fig. 3a. The amount of nanofibers increased significantly when reaction time increased to 6 h. Furthermore as the reaction time increased to 12 h, the nanofibers became dominant. Figure 3e shows a TEM image of BaTiO_3_ NFs synthesized at 210 °C for 12 h. It can be observed that the surface of BaTiO_3_ NFs was rough and a HRTEM image of a typical BaTiO_3_ NF synthesized at 210 °C for 12 h is shown in Fig. 3f. The clear lattice fringes illustrated that the BT NF were single-crystalline. The parallel lattice spacing were about 0.406 nm and 0.286 nm corresponding to (001) and (101) planes of tetragonal BaTiO_3_ respectively, which revealed that the NFs grew in the [001] direction. The corresponding selected area diffraction pattern (SADP) also exhibited the characteristics of a single crystal.

**Figure 4:**
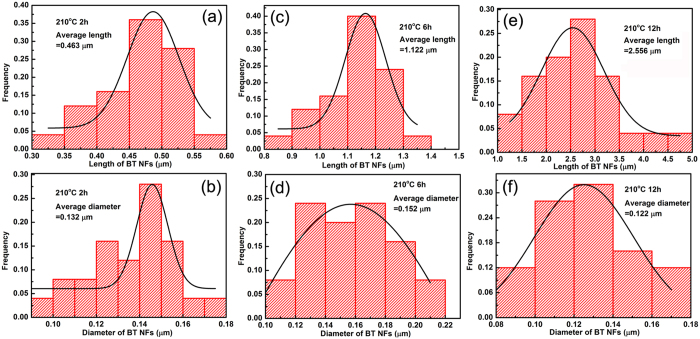
BaTiO_3_ NFs length and diameter distribution for different reaction times. (**a**,**b**) 210 °C. 2 h. (**c**,**d**) 210 °C. 6 h. (**e**,**f**) 210 °C, 12 h.

It was worth noting that with an increase of reaction time, the length of the BaTiO_3_ NFs increased to a much greater extent compared to the diameter, leading to an increase in the fibre aspect ratio. The length and diameter of the BaTiO_3_ NFs synthesized at 210 °C for 2 h, 6 h and 12 h were analyzed using SEM images by ImageJ software, as shown in Fig. 4. The aspect ratio of the BaTiO_3_ NFs calculated from Fig. 4 were 3.5, 7.4 and 21.0 for the reaction time of 2 h, 6 h and 12 h, respectively and clearly shows that the aspect ratio of the BaTiO_3_ NFs increased with an increase of reaction time.

**Figure 5:**
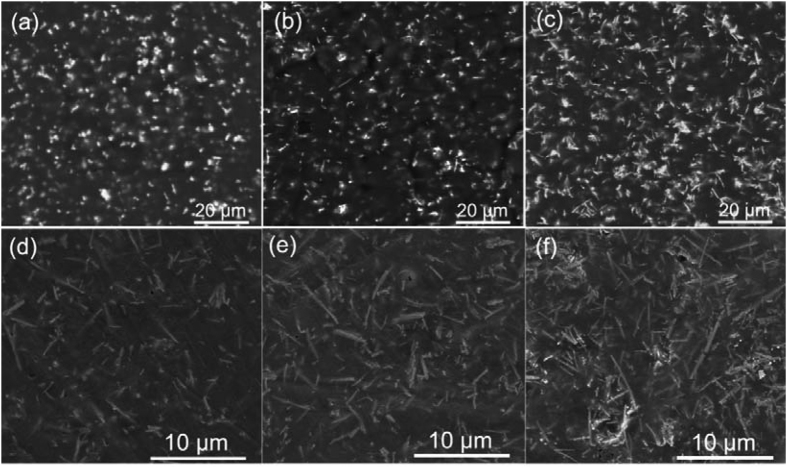
(**a**–**c**) SEM images of the nanocomposites with 5 vol% low aspect ratio BaTiO_3_ NFs synthesized at 210 °C for 2 h, 6 h and 12 h. (**d**–**f**) SEM images of the nanocomposites with 2.5 vol%, 5.0 vol% and 7.5 vol% high aspect ratio BaTiO_3_ NFs synthesized at 210 °C for 12 h.

To improve the compatibility and dispersibility of BaTiO_3_ NFs in the P(VDF-HFP) matrix, the BaTiO_3_ NFs were surface functionalized by dopamine. As highlighted in the introduction, the breakdown strength of the nanocomposite is equally important as the dielectric constant with regard to the energy density and the breakdown strength tends to decrease with increasing volume fraction of ceramic filler. In order to improve the dielectric properties of nanocomposites and maintain a high breakdown strength, the nanocomposites were fabricated at a relatively low volume fraction of BaTiO_3_ NFs in this study, varying from 2.5% to 7.5%. The upper-surface morphology of the BaTiO_3_ NFs/P(VDF-HFP) nanocomposites are shown in Fig. 5. Figure 5a–c shows SEM images of the nanocomposites with 5.0 vol% of BaTiO_3_ NFs synthesized at 210 °C for 2 h, 6 h and 12 h, respectively where it can be seen that the aspect ratio of the BaTiO_3_ NFs in nanocomposites gradually increased. Figure 5d–f shows the SEM images of the nanocomposites with 2.5 vol%, 5.0 vol% and 7.5 vol% BaTiO_3_ NFs synthesized at 210 °C for 12 h. The visibility of the BaTiO_3_ NFs in nanocomposite became more pronounced with increasing BaTiO_3_ NFs volume fraction. It can be observed that the BaTiO_3_ NFs exhibited good compatibility and dispersibility in the P(VDF-HFP) matrix and the nanocomposites exhibited limited defects as the volume fraction of BaTiO_3_ NFs approached 7.5%. This can be attributed to that the fact that the BaTiO_3_ NFs were surface modified by dopamine and therefore formed a strong adhesive bonding force with the polymer matrix.

**Figure 6:**
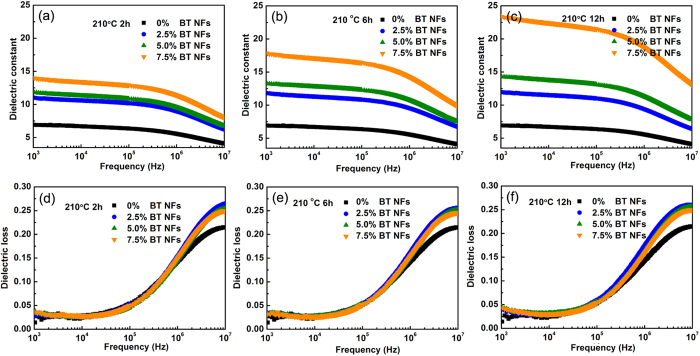
Dependency of dielectric constant and loss of the nanocomposites on the aspect ratio (AR) and volume fraction of BT NFs with frequency ranging from 1 kHz to 10 MHz. (**a**,**d**) 210 °C 2 h; AR = 3.5, (**b**,**e**) 210 °C 6 h; AR = 7.4, (**c**,**f**) 210 °C 12 h; AR = 21.0.

Figure 6 shows the variation of the dielectric constant and loss of the nanocomposites with the BaTiO_3_ NFs aspect ratio and volume fraction for a frequency range of 1 kHz to 10 MHz. The dielectric constant of the nancomposites increased with the increase of volume fraction of BaTiO_3_ NFs since BaTiO_3_ possess a higher dielectric constant compared with pure P(VDF-HFP) polymer. At the measurement frequency of 1 kHz, the dielectric constant of the nanocomposite with 7.5 vol% BaTiO_3_ NFs synthesized at 210 °C for 12 h (aspect ratio 21.0) reached 23.4 while the dielectric constant of the samples with 7.5 vol% BaTiO_3_ NFs synthesized at 210 °C for 6 h (aspect ratio 7.4) and 2 h (aspect ratio 3.5) was 17.8 and 14 respectively. This demonstrates that on increasing the aspect ratio of the BaTiO_3_ NFs, the dielectric constant of the nancomposites was significantly improved. There are a number of previous reports and theoretical models that have demonstrated the increased dielectric constant as a result of using high aspect ratio ceramic fillers^14^,^25^,^26^. For instance, the Maxwell-Garnet model can efficiently describe the effect of aspect ratio, as shown in equation 1^35^,^36^.


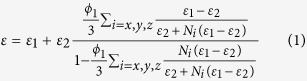


where N_*i*_ is known as the depolarization factor of ellipsoids in the x, y, z direction. For BaTiO_3_ NFs, where the radii a_*x*_ > a_*y*_ = a_*z*_. N_*i*_ can be expressed as equation 2


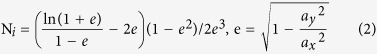


The Maxwell-Garnet model indicates that the dielectric constant of the nanocomposites will increase on increasing the aspect ratio of BaTiO_3_ NFs.

However, there was no apparent variation about the dielectric loss on increasing the aspect ratio of BaTiO_3_ NFs. For example, the dielectric loss of the nanocomposites with 7.5 vol% BaTiO_3_ NFs synthesized at 210 °C for 2 h, 6 h and 12 h was 0.032, 0.037 and 0.046 at 1 kHz, respectively. The dielectric loss remained less than 0.06 (<100 kHz) for all samples which was attributed to the relatively low volume fraction of BaTiO_3_ NFs and their good compatibility and dispersion in the polymer matrix. The dielectric properties of nanocomposites were efficiently improved by increasing the aspect ratio of the BaTiO_3_ NFs, without the need for additional fillers.

**Figure 7:**
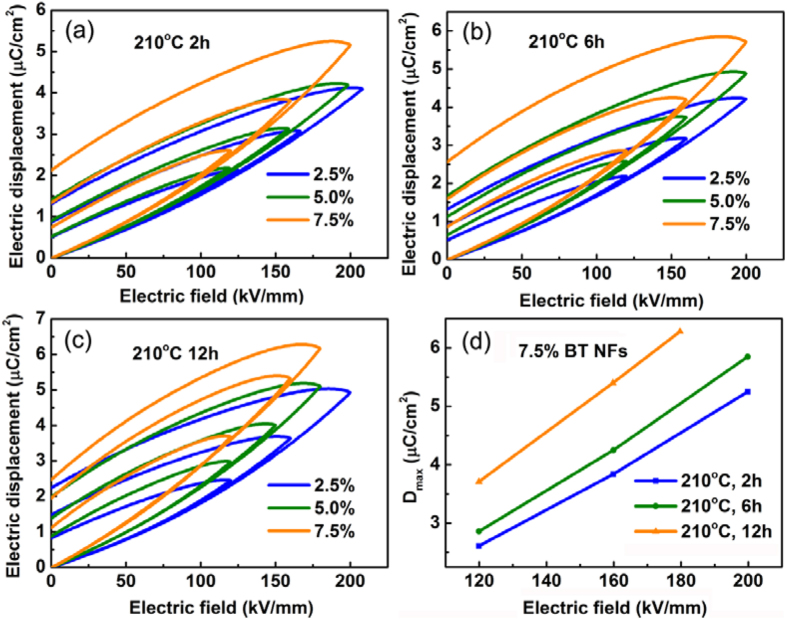
The D-E loops of nanocomposites where the volume fraction of BaTiO_3_ NFs ranged from 2.5% to 7.5% and the aspect ratio (AR) of BaTiO_3_ NFs was (**a**) 210 °C 2 h; AR = 3.5, (**b**) 210 °C 6 h; AR = 7.4, and (**c**) 210 °C 12 h; AR = 21.0. (**d**) The D_max_ of the nanocomposites with electric field for 7.5 vol% BT NFs with various aspect ratios.

Figure 7a–c show typical electric displacement-electric field (D-E) loops of nanocomposites where the volume fraction of BaTiO_3_ NFs ranged from 2.5% to 7.5% and the aspect ratio of the BaTiO_3_ NFs were 3.5, 7.4 and 21.0 respectively. As can be seen from Fig. 6a, the maximum electric displacement (D_max_) increased monotonically with an increase of the volume fraction of BaTiO_3_ NFs. A similar trend can also be observed in Fig. 6b and c. Figure 6d summarizes the D_max_ of nanocomposites with 7.5 vol% for various aspect ratios of BaTiO_3_ NFs and the D_max_ was enhanced with increasing the electric field. Figure 7d also clearly demonstrated that with increasing the aspect ratio of BaTiO_3_ NFs, the D_max_ of nanocomposites improved significantly. For instance, the D_max_ of the nanocomposites with 7.5 vol% and higher aspect ratio (AR) BaTiO_3_ NFs (AR = 21) reached 5.4 μC/cm^2^ while the value was only 3.9 μC/cm^2^ for lower aspect ratio BaTiO_3_ NFs (AR = 3.5) under an electric field of 160 kV/mm. It has been demonstrated in Fig. 6 that the dielectric constant of nanocomposites increases on increasing the aspect ratio of the BT NFs. Since D = ε_0_ε_r_E, where ε_0_ and ε_r_ are free space and the relative of the permittivity of the nanocomposites respectively, then under the same electric field (E) an increase in D_max_ can result from an increase of dielectric constant of the nanocomposites on increasing the volume fraction or aspect ratio of the BaTiO_3_ NFs.

**Figure 8:**
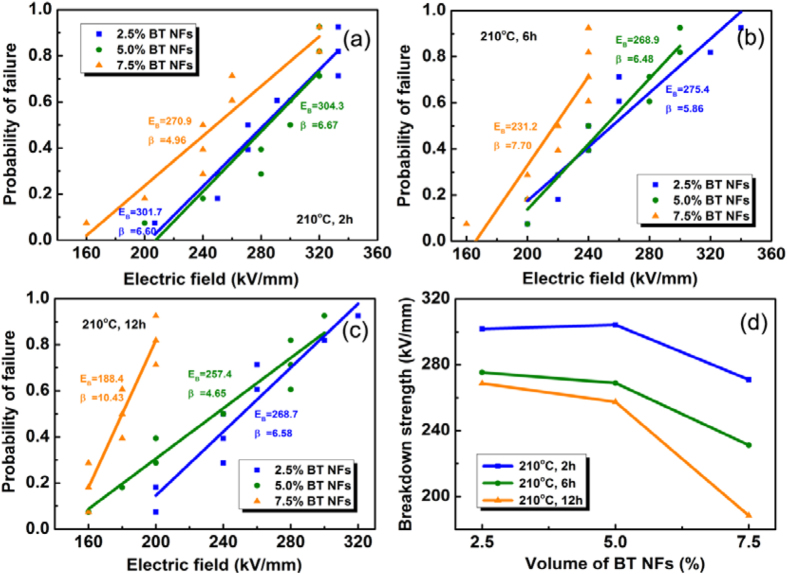
The electric breakdown strength of nanocomposites with different volume fraction and aspect ratio (AR) BaTiO_3_ NFs. (**a**) 210 °C 2 h; AR = 3.5, (**b**) 210 °C 6 h; AR = 7.4, (**c**,**f**) 210 °C 12 h; AR = 21.0.

The electric breakdown strength of the nanocomposites was analyzed by a two parameter Weibull cumulative probability function, as shown in equation 3,


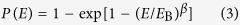


where *P(E*) is the cumulative probability of electric failure, *E* is the experimental breakdown strength, *E*_B_ is a scale parameter that refers to the breakdown strength at the cumulative failure probability of 63.2% which is regarded as the characteristic breakdown strength, and *β* is the Weibull modulus associated with the linear regressive fit of the distribution. Figure 8a–c shows the electric breakdown strength of nanocomposites with different volume fraction and aspect ratio BaTiO_3_ NFs. The characteristic breakdown strength (E_B_) is summarized in Fig. 7d for the different volume fractions and aspect ratios. All samples possessed a relatively high breakdown strength over 180 kV/mm and on increasing the volume fraction of BaTiO_3_ NFs, the E_B_ of the nanocomposites decreased; see Fig. 8d. The E_B_ of the nanocomposites with 2.5 vol% BaTiO_3_ NFs and aspect ratio of 21 was 268.7 kV/mm, which reduced to 257.4 kV/mm and 188.4 kV/mm when the volume fraction of BaTiO_3_ NFs increased to 5.0% and 7.5%, respectively. The introduction of more BaTiO_3_ NFs into the polymer matrix resulted in defects and electric field concentrations, which increased the risk of failure and decreased the breakdown strength. The nature of the field concentrations with respect to volume fraction and aspect ratio are discussed later. It also can be demonstrated in Fig. 8d that the E_B_ of the nanocomposites decreased with an increase of the aspect ratio of the BaTiO_3_ NFs.

The energy storage density of the nanocomposites were calculated from D-E loops according to the equation 4,


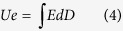


**Figure 9:**
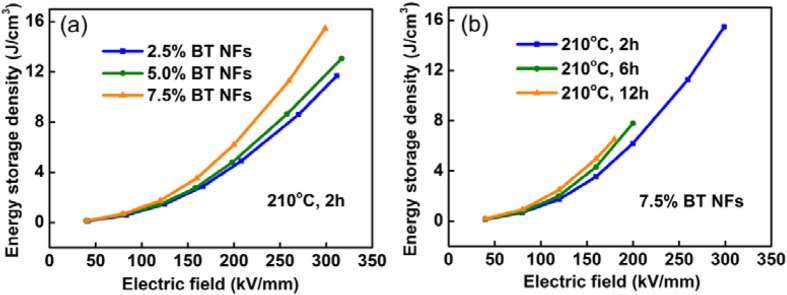
(**a**) Energy storage density of the nanocomposites with BaTiO_3_ NFs synthesized at 210 °C for 2 h (aspect ratio 3.5) as a function of electric field and volume fraction. (**b**) Energy storage density of the nanocomposites with 7.5 vol% BaTiO_3_ NFs as a function of electric field and aspect ratio (AR): 210 °C 2 h (AR = 3.5), 210 °C 6 h (AR = 7.4), 210 °C 12 h (AR = 21.0).

Figure 9a presents the energy storage density of the nanocomposites with low aspect ratio BaTiO_3_ NFs synthesized at 210 °C for 2 h as a function of electric field and volume fraction of BaTiO_3_ NFs. This indicates that the energy storage density increased with an increase of electric field. Moreover, the nanocomposites with the higher volume fraction of BaTiO_3_ NFs possessed the higher energy storage density under the same electric field. Figure 9b shows the energy storage density of the nanocomposites with 7.5 vol% BaTiO_3_ NFs as a function of electric field and aspect ratio of the BaTiO_3_ NFs. Similarly, the nanocomposites with the higher aspect ratio BaTiO_3_ NFs exhibited a higher energy storage density under the same electric field. The higher energy storage density can be attributed to the significantly enhanced dielectric constant and D_max_ of the nanocomposites on increasing the volume fraction and aspect ratio of the BaTiO_3_ NFs. Figure 9b also indicates that the electric breakdown strength of the nanocomposites decreased with increasing aspect ratio of BaTiO_3_ NFs. Under an electric field of 300 kV/mm, the maximal discharged energy density of 15.48 J/cm^3^ was obtained in the nanocomposites with 7.5 vol% on low aspect ratio BaTiO_3_ NFs synthesized at 210 °C for 2 h.

To provide a deeper understanding of the decrease in breakdown strength and increase in dielectric constant with an increase in BaTiO_3_ NF volume fraction and aspect ratio, finite element modelling (Ansys APDL v15.0) was employed. A two dimensional electrostatic analysis of a single high permittivity BaTiO_3_ NF (relative permittivity, ε_r_ = 1500) embedded in a low permittivity P(VDF-HFP) matrix (ε_r_ = 7) was used to investigate the effect of fiber aspect ratio and angle (with respect to applied field direction) on the maximum localized electric field. BaTiO_3_ composites in the range of the experimentally produced aspect ratios (3.5 to 20.1) and volume fractions (2.5 vol.% to 7.5 vol.%) were analyzed.

**Figure 10:**
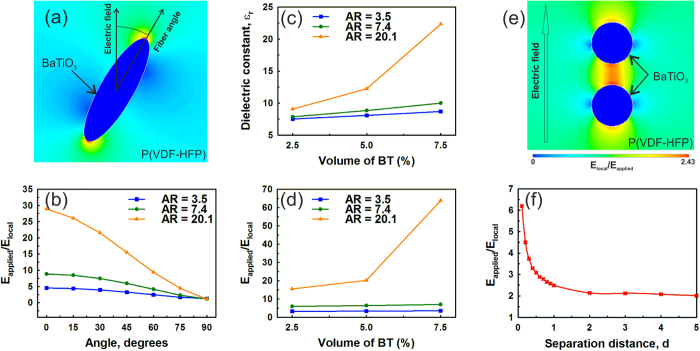
(**a**) Contour plot of finite element analysis for BaTiO_3_ fiber (2.5 vol.%) in P(VDF-HFP) matrix with aspect ratio 3.5 orientated at 30° to the applied field, where blue and red contours represent regions of high and low field, respectively; (**b**) effect of fiber angle and aspect ratio on maximum local field concentration (plotted as E_local_/E_applied_); (**c**,**d**) show the effect of BaTiO_3_ fiber aspect ratio and volume fraction at a 45° angle to applied field on dielectric constant and maximum local field concentration, respectively; (**e**) contour plot of two BaTiO_3_ NFs aligned in field direction, showing field concentration between particles; (**f**) effect of separation distance on field concentration within P(VDF-HFP) matrix.

The inclusion of a high permittivity BaTiO_3_ inclusion leads to the electric field concentrating in the low permittivity matrix, and such field concentrations are likely to be sites for initiating dielectric breakdown within these nanocomposites. This can be seen in Fig. 10a, which is a close up image of the field distribution around a BaTiO_3_ fiber with an aspect ratio of 3.5 at 2.5 vol.%. The blue contours are low field regions and red contours indicate high field concentrations at the tips of the fiber. High aspect ratio fibers aligned almost perpendicular to the applied field direction (angle > 85°) produced slightly lower electric field concentrations compared to lower aspect ratios, however at angles below 85° the high aspect ratio fibers result in local electric fields that are significantly higher than the applied field, see Fig. 10b. The manufactured composites had a random orientation of fibers within the film (Fig. 5) and therefore a nominal fiber orientation of 45° to the applied field was used to demonstrate the effect of BaTiO_3_ aspect ratio and volume fraction on the relative permittivity (dielectric constant) and field concentration, where an increase in local electric field leads to a reduced breakdown strength. The relative permittivity (Fig. 10c) and localized field concentrations (Fig. 10d) were found to increase with an increase in both fiber aspect ratio and volume fraction. These results agree well with the experimental observations that on increasing the aspect ratio and volume fraction of the BaTiO_3_ NFs, the dielectric constant of the nanocomposites was improved (Fig. 6a–c) while the breakdown strength of the nanocomposites decreased (Fig. 8d).

High BaTiO_3_ NF aspect ratios are also likely to reduce the average separation distance between high permittivity inclusions for the same volume fraction. A finite element model of two BaTiO_3_ particles (aspect ratio of unity) within a P(VDF-HFP) matrix was used to demonstrate the effect of separation distance on electric field concentrations within the matrix. The ‘worst-case’ in terms of field concentration was occurred when two particles were aligned in the direction of the field, as in Fig. 10e. The ratio of the maximum local field to the applied field is shown in Fig. 10f as a function of the distance between the high permittivity particles, with the electric field concentration following an inverse separation law. These result indicate that a combination of electric field concentrations due to the presence of high permittivity fibers and fiber separation distance influence the permittivity and dielectric strength and the high aspect ratio fibers lead to a lower breakdown strength, as observed experimentally (Fig. 8d).

## Conclusion

In this study, BaTiO_3_ nanofibers (BT NF) with a variety of aspect ratios were synthesized by a two-step hydrothermal method. The effects of the aspect ratio and volume fraction of the BT NF on dielectric properties and energy storage densities of the P(VDF-HFP) based one-dimensional nanocomposites were investigated and modeled in detail. As the aspect ratio and volume fraction of the BaTiO_3_ NFs was increased, the dielectric constant and D_max_ of the nanocomposites were both increased monotonically while the breakdown strength decreased. The nanocomposites with highest aspect ratio and volume fraction of BT NFs exhibited the highest energy storage density under the same electric field. The maximal energy storage density reached 15.48 J/cm^3^ in the nanocomposites with 7.5 vol% BT NFs synthesized at 210 °C for 2 h under the electric field of 300 kV/mm. This work provides a potential new route to prepare and tailor the properties of novel high energy density capacitor nanocomposites.

## Methods

### Synthesis of BaTiO_3_ nanofibers

The BaTiO_3_ nanofibers (BT NFs) were synthesized by a two-step hydrothermal method. Firstly, sodium titanate nanofibers (Na_2_Ti_3_O_7_ NFs, NT NFs) were synthesized. A 1.446 g mass of titanium oxide (TiO_2_, Anatase) was added to 70 ml NaOH solution (10 M) and the mixture was stirred for 2 h to form a homogeneous suspension. Hydrothermal reactions were carried out at 210 °C under an auto-generated pressure for 24 h in a 100 ml Teflon-lined autoclave. The products were washed by distilled water and then soaked in diluted 0.2 M hydrochloric acid (HCl, 37%) for 4 hours to obtain hydrogen titanate nanofibers (H_2_Ti_3_O_7_ NFs). The BT NFs were synthesized by a second hydrothermal reaction where 0.150 g of H_2_Ti_3_O_7_ NFs were dispersed in 70 ml Ba(OH)_2_∙8H_2_O solution and the mixture was sonicated for 10 min. The hydrothermal reactions were carried out at 210 °C under an auto-generated pressure for 2–24 h in a 100 ml Teflon-lined autoclave to obtain BT NFs at a range of aspect ratios. The products were soaked in 0.2 M HCl solution briefly, then washed using distilled water several times and dried at 80 °C in an oven.

### Fabrication of BT NFs/P(VDF-HFP) nanocomposite

The BT NFs were mixed with a solution of P(VDF-HFP) in *N,N*-dimethylformamide (DMF) by stirring and sonicating to form a homogeneous suspension. The suspension was then cast onto a clean glass and dried at 80 °C for 12 h under vacuum. The dried nanocomposite sheets were then compressed into films at 200 °C under a pressure of approximately 15 MPa. Gold electrodes were sputtered on both sides of the film using a mask with 2 mm diameter eyelets.

### Characterization

The crystalline phases of the NFs were evaluated by X-ray diffraction (XRD, D/max 2550, Japan) with Cu-Kα radiation (λ = 1.5406 Å) at room temperature. The size and morphology of the NFs and the nanocomposites were observed using a scanning electron microscope (SEM, Nova NanoSEM230, USA). High-resolution transmission electron microscopy (HRTEM) images of the NFs were taken with a Titan G2 60–300 instrument, using an accelerating voltage of 300 kV. The frequency-dependent dielectric constant and dielectric loss were measured using an Agilent 4294 A LCR meter with frequency ranging from 1 kHz to 10 MHz. The electric displacement-electric field loops and leakage current were measured by a precision Premier II ferroelectric polarization tester (Radiant, Inc.) at room temperature and 10 Hz.

## Additional Information

**How to cite this article:** Zhang, D. *et al*. Significantly Enhanced Energy Storage Density by Modulating the Aspect Ratio of BaTiO_3_ Nanofibers. *Sci. Rep.* **7**, 45179; doi: 10.1038/srep45179 (2017).

**Publisher's note:** Springer Nature remains neutral with regard to jurisdictional claims in published maps and institutional affiliations.
