# Author Correction: Significantly Enhanced Energy Storage Density by Modulating the Aspect Ratio of BaTiO_3_ Nanofibers

**DOI:** 10.1038/s41598-020-65656-z

**Published:** 2020-05-26

**Authors:** Dou Zhang, Xuefan Zhou, James Roscow, Kechao Zhou, Lu Wang, Hang Luo, Chris R. Bowen

**Affiliations:** 1 State key Laboratory of Powder Metallurgy, Central South University, Changsha, Hunan 410083 China; 2 Department of Mechanical Engineering, University of Bath, Bath, BA2 7AY UK; 3 College of Chemistry and Chemical Engineering, Central South University, Changsha, 410083 China

Correction to: *Scientific Reports* 10.1038/srep45179, published online 23 March 2017

This article contains errors in Figure 5. The SEM images of the nanocomposites with 2.5 vol%, 5.0 vol% and 7.5 vol% BaTiO_3_ NFs synthesized at 210 °C for 12 h shown in panels d-f were incorrectly given as SEM images of P(VDF-HFP) based nanocomposites with 5.0 vol%, 10.0 vol% and 20.0 vol% BaTiO_3_ NFs synthesized at 210 °C for 24 h. The correct Figure 5 appears below as Fig. 1.

**Figure 1:**
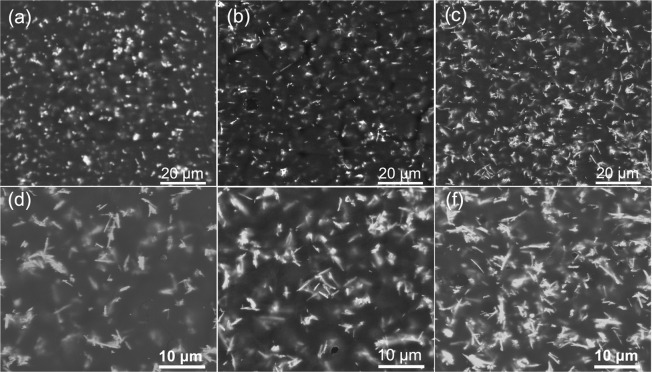
(**a–c**) SEM images of the nanocomposites with 5 vol% low aspect ratio BaTiO3 NFs synthesized at 210 °C for 2 h, 6 h and 12 h. (**d–f**) SEM images of the nanocomposites with 2.5 vol%, 5.0 vol% and 7.5 vol% high aspect ratio BaTiO3 NFs synthesized at 210 °C for 12 h.

